# Exploring the barriers and enablers experienced by people with Cystic Fibrosis and their healthcare professionals in accessing, utilising and delivering maternity and Cystic Fibrosis care during the pre-conception to post-partum period: A mixed methods systematic review protocol.

**DOI:** 10.12688/hrbopenres.13500.2

**Published:** 2023-04-28

**Authors:** Jen Balfe, Jennifer Donnelly, Sarah Tecklenborg, Aisling Walsh

**Affiliations:** 1 RCSI University of Medicine and Health Sciences, Dublin, Ireland; 2 Rotunda Hospital, Dublin, Ireland; 3 Mater Misericordiae University Hospital, Dublin, Ireland; 4 University College Dublin, Dublin, Ireland; 5 Cystic Fibrosis Ireland, Dublin, Ireland

**Keywords:** Cystic Fibrosis, pregnancy, maternity, barriers, enablers, healthcare professionals, women with Cystic Fibrosis, systematic review.

## Abstract

Background

Cystic Fibrosis (CF) is an autosomal recessive inherited multi-system disease that primarily affects the lungs and digestive system. New drug therapies and treatments are improving the lives of many people with CF. With improved life expectancy and increased quality of life, many people with CF are now contemplating parenthood and becoming pregnant, an aspiration that decades ago was almost unheard of. Given this quickly evolving and more positive health landscape, it is vital to understand how people with CF experience the care they receive whilst accessing and utilising fertility and maternity services. It is also important to explore the experiences of healthcare professionals involved in providing care during this period. The overall aim of the mixed-methods systematic review will be to explore the barriers and enablers experienced by people with CF and the healthcare professionals involved in their care in the pre-conception to post-partum period.

Methods

The proposed review will be conducted in accordance with the Joanna Briggs Institute (JBI) methodology for convergent integrated mixed methods systematic reviews. A systematic search of Medline (Ebsco), Cinahl, Embase, APA PsychINFO and Cochrane Library from inception to February 2022 will be conducted. Quantitative, qualitative and mixed methods studies pertaining to the experience of pre-conception to post-partum care for people with CF and their healthcare professionals will be included. Two independent reviewers will screen titles, abstracts and full texts with disagreements being resolved by a third reviewer.

Conclusion

This review will help to determine the potential barriers and facilitators experienced by people with Cystic Fibrosis and the health care professionals involved in their care during the pre-conception to post-partum period. The results will be of benefit specifically to the CF population and their healthcare providers when planning further studies in the area of fertility and pregnancy for this population and when delivering care.

## Introduction

Cystic Fibrosis (CF) is an autosomal recessive, inherited multi-system disease that primarily affects the lungs and digestive system. Once considered a disease of childhood, improved therapies and better management has led to a changed demographic in the CF population, and many adults with CF are now experiencing improved quality and quantity of life ^1, 2^.

The first reported incident of pregnancy in a person with Cystic Fibrosis (CF) was in 1960 ^3^. The deterioration of the patient two months before delivery and her death soon after, established pregnancy as an undesirable and dangerous path for patients with CF. At a time where treatment options were limited and CF was primarily seen as a paediatric illness, the concept of pregnancy within this context as being an unfavourable complication is unsurprising. Much of the discourse in the following decades has focused on the maternal outcomes in pregnancies of people with CF (pwCF), the potential risks and complications of pregnancy in CF as well as guidelines for the management of patients with CF in pregnancy ^4– 6^.

With improved care and the advent of Cystic Fibrosis transmembrane conductance regulator (CFTR) modulator therapies, the landscape for many pwCF has changed dramatically, meaning that pregnancy and parenthood are now achievable aspirations. In a survey of 188 young women with CF in the US, seventy-eight percent expressed a desire to have children in the future ^7^. This is in fact evidenced in recent years as many countries have seen increases in the numbers of pregnancies being recorded in international CF registries ^1, 2^.

Recent data suggest that pregnancy is now a considered choice for women with CF and they give consideration to the multi-factorial impacts the decision to conceive may have on their health status and wellbeing ^8^, even before addressing it with their healthcare providers ^9^. It is therefore essential to assess how this relatively recent phenomenon is experienced by people with CF and the healthcare professionals involved in their care in order to best provide care in the future.

Much of the literature that exists on pregnancy and CF is weighted in terms of outcomes and guidelines and is focused on the physiological and medical implications that CF has on maternal and foetal outcomes, or in terms of the effects pregnancy has on CF baseline parameters - such as pulmonary function ^5, 6, 10– 12^. While these studies often highlight how pregnancy in the main is well tolerated by pwCF, analysis of the experience of pregnancy by this population needs further investigation.

With a changing reality, more people with CF are navigating this new terrain and desire open communication around issues of sexual and reproductive health and pregnancy decision making in CF. A study on contraceptive options in CF, notes the high rates of unintended pregnancy in women with CF ^13^, and another on reproductive health in CF asserts the need for improved communication as means of facilitating better decision making pre-conception ^14^. Hughan postulates that ‘Reproductive health decision-making tools should be explored as a way to enhance patient-provider communication surrounding reproductive health topics’ ^15^. A number of studies highlight how women with CF seek up to date and reliable information when planning a pregnancy or whilst pregnant ^10, 11^ especially in relation to the management and potential contra indications of their medications as well as advice on treatments and adherence ^16^.

The importance of collaborative care between CF and high-risk obstetrical care teams was highlighted in a recent review ^17^. The lack of detailed information, collected by the large CF registries on delivery and foetal outcomes was also highlighted in this study ^17^.

Analysing and synthesising the lived and evolving experience of pwCF and their healthcare professionals of pre-conception to post-partum care and identifying the barriers both groups encounter during this period will help to elucidate areas which require attention and future planning. Identifying enablers that add to the experience in a positive and practical way will also add to the growing bank of recommendations for health service provision in the future ^18^. Comparing the experiences of both groups will also serve to highlight any gaps that may exist in how each group experiences accessing, utilising or delivering care in the pre-conception to post-partum period.

## Review question

The mixed methods systematic review will seek to answer what are the barriers and enablers encountered by people with CF and the healthcare professionals involved in their care in accessing, utilising and delivering pre-conception, antenatal, intra-partum and post-partum care (pre-conception to post-partum care)? This will be addressed through two objectives; i) to explore the experiences of people with Cystic Fibrosis while accessing and utilising pre-conception to post-partum care; ii) to explore the experiences of healthcare professionals involved in providing fertility, obstetric and Cystic Fibrosis care to people with CF in the pre-conception to post-partum period.

## Inclusion criteria

### Population

The review will consider studies that include people with CF who have experienced any or all stages of maternity care which we define as including, pre-conception, antenatal, intra-partum and post-partum care. The review will also consider studies that include healthcare professionals involved in delivering care, be that CF specific care or fertility and obstetric care to people with CF during this time.

### Phenomena of interest

The experience under study for this review will be that of fertility, pregnancy, delivery and postnatal care in people with Cystic Fibrosis. The review will consider studies that investigate the pre-conception experience to post-partum experience for people with CF and health professionals that provide care to people with CF during this time.

Barriers will be identified as any perceived or experienced construct, attitude, or challenge, either systemically, internally or otherwise that impedes the experience of accessing, utilising or delivering, pre-conception to post-partum care. Enablers will be identified as any perceived or experienced facilitator of positive experience in accessing, utilising or delivering pre-partum to post-partum care. Approaching the concepts of barriers and enablers through a macro, meso and micro lens will also enable the identification of challenges and facilitators as they arise at different levels. This strategy was employed by Smith et al in an Australian study which sought to identify barriers and enablers which affected extended scopes of practice in rural Australia in relation to nurse practitioners. As such this lens may prove a suitable approach in identifying barriers and enablers as experienced by pwCF and healthcare professionals (HCPs) ^19^. Although focusing on the HCP perspective the authors provide an adaptable and employable set of definitions for the three stages that will be addressed in this study and barriers and enablers will be identified and adapted using the framework employed by Smith *et al.*.

### Context

Given the incidence of pregnancy is rising in this population internationally ^1, 2^, all geographical locations and settings will be considered. Settings may include but are not limited to; hospitals, clinics, community care or primary healthcare settings. Settings will be included if they are identified as providing fertility, obstetric, maternity or Cystic Fibrosis care to pwCF in the pre-conception to post-partum period.

### Types of studies

The review will consider quantitative, qualitative and mixed-methods studies (if it is possible to clearly extract the quantitative and qualitative components of such studies).

Qualitative studies will include designs such as descriptive qualitative design, phenomenology, grounded theory, ethnography, participatory and action research, and feminist research. This will include interviews, focus group discussions, ethnographic data and journaling that explore the experiences of pwCF and their HCPs during the pre-conception to post-partum period.

Quantitative studies will include observational studies (e.g. cohort studies, cross-sectional studies and case-control studies). The quantitative component of this review will consider studies that include questionnaires that are aimed at ascertaining the experience of pwCF or HCPs in accessing, utilising or providing care during the pre-conception to post-partum period. Such studies may report sociodemographic data which may help to provide a broader picture about how sociodemographic factors can influence the experience of accessing, utilising or delivering care in pwCF during the pre-conception to post-partum period.

The qualitative and quantitative components of this review will consider studies that investigate how the barriers and enablers perceived or encountered by people with CF and their healthcare professionals during the pre-conception to post-partum period, contribute to their experience of accessing, utilising and delivering care.

To correspond with the first reporting of a case of pregnancy in a person with CF in 1960, studies published from this year to the present will be included.

## Methods

The review will be conducted in accordance with the JBI methodology for mixed methods systematic reviews, following a convergent integrated approach ^20^. This approach suggests that when a review question is answerable by both quantitative and qualitative data that ‘an integrated approach to synthesis is undertaken’ and data transformation should be employed ^20^.

Quantitative and qualitative data which presents or explores the experiences of pwCF and healthcare professionals in accessing, utilising and delivering fertility, maternity and CF care during the pre-conception to post-partum period will be examined in order to ascertain any perceived or experienced barriers and enablers relating to the above mentioned period.

A preliminary search of Medline and Cinahl has been undertaken and to the best of our knowledge no existing or ongoing mixed method or individual systematic reviews on the topic have been identified.

### Search strategy and Information Sources

An initial limited search of MEDLINE and CINAHL will be undertaken to identify articles on the topic and to inform the search strategy. A librarian from the Royal College of Surgeons in Ireland has assisted in the development of the search strategy. The text words contained in the titles and abstracts of relevant articles, and the index terms used to describe the articles will be used to develop a full search strategy for Medline, Cinahl, Embase, APA PsycInfo and Cochrane Library. The search strategy, including all identified keywords, and index terms relating to barriers and enablers in accessing, utilising and delivering pre-conception to post-partum care for pwCF will be adapted for each included information source. The reference list of all studies selected for critical appraisal will be screened for additional studies.

### Study selection

Following the search, all identified citations will be loaded into EndNote X9 and duplicates removed. Titles and abstracts will then be screened by two independent reviewers for assessment against the inclusion criteria for the review using Covidence software. Potentially relevant studies will be retrieved in full and their citation details imported into Covidence. The full text of selected citations will be assessed against the inclusion criteria by two independent reviewers. Reasons for exclusion of full text studies that do not meet the inclusion criteria will be recorded and reported in the systematic review. Any disagreements that arise between the reviewers at each stage of the study selection process will be resolved through discussion, or with a third reviewer. The results of the search will be reported in full in the final report and presented in a Preferred Reporting Items for Systematic Reviews and Meta-analyses (PRISMA) flow diagram ^21^.

### Assessment of methodological quality

Quantitative studies selected for retrieval will be assessed by two independent reviewers for methodological validity prior to inclusion in the review using standardised critical appraisal instruments from JBI ^22^.

Qualitative studies selected for retrieval will be assessed by two independent reviewers for methodological validity prior to inclusion in the review using the standardised critical appraisal instrument from JBI ^23^.

Mixed methods studies will be appraised using MMAT, a mixed methods critical appraisal tool ^24^.

Authors of papers will be contacted to request missing or extra data for clarification purposes, where required. If disagreements arise between the reviewers, they will be resolved through discussion, or with a third reviewer. To capture the experience of both people with CF and HCPs, all studies, regardless of the results of their methodological quality, will undergo data extraction and synthesis (where possible). The results of the critical appraisal will be reported in narrative form and in a table.

### Data extraction

Quantitative and qualitative data will be extracted from studies included in the review by two independent reviewers, using Covidence software. 

The data extracted will include details on both populations identified in the study. In the case of HCPs, details on the specific area of care they are associated with, i.e. CF specific care or obstetric care etc. Further data on the specific role within the care team will also be identified, i.e. Consultant, CF Nurse, Midwife, Psychologist etc. In terms of the data regarding pwCF, details on age, gender and service accessed or utilised will be included where available. Details on study methods and design, and results relating to experience of accessing/utilising or providing pre-conception to post-partum care will be recorded. Details on barriers/enablers as experienced/perceived by pwCF or HCPs will also be recorded. Details on specific settings will be noted, including but not limited to; fertility clinic, maternity hospital, primary care centre, CF centre, and acute hospital. Details on the particular stage of care (pre-conception, ante-natal, intra- partum and postpartum), and geographical location will also be recorded. Main results, recommendations and evidence gaps will also be extracted.

Data extracted from quantitative studies will include any data-based outcomes relating to the experience of pwCF and the HCPs involved in the care of pwCF during the specified period. Qualitative data will be assessed and categorised by theme and sub-theme relating to the review question and a quotation from the direct quote or observation from the study in question will be provided. The sample data extraction chart is included and will be refined following piloting in 2023. A sample of five articles which have progressed to full text screening will be piloted by two reviewers. 

The lead author will review all articles and two other reviewers will sample half the texts each. The data extraction template will be completed by two reviewers. The piloted extraction sheets will be reviewed by the two reviewers and any conflicts will be resolved by consensus or by decision of a third reviewer.

### Data synthesis and integration

The review will follow a convergent integrated approach according to the JBI methodology for mixed methods systematic reviews using JBI SUMARI ^20^.

Once data extraction is complete the quantitative data will be transformed into ‘qualitised’ data, whereby textual descriptions and narrative interpretation will be used to transform the quantitative data. This will enable integration with data extracted from the qualitative studies and the qualitative components of mixed methods studies ^20^. This particular approach involves gathering the qualitised data with the qualitative data. This assembled data will then be categorised and pooled together to create a set of integrated findings.

### Study status

At the time of publication of this mixed method systematic review protocol, search terms have been piloted and are currently being refined. Full database searches will be run in February 2022.

## Discussion

The changing landscape of maternal health in the context of Cystic Fibrosis care is worthy of exploration. Though now not an uncommon experience as once it was, it is vital that we understand how the experience of accessing, utilising and delivering all aspects of maternal care is experienced by pwCF and their HCPs. A systematic review of the literature pertaining to the lived experiences of pwCF and their HCPs will illuminate the current barriers and enablers as experienced by both groups and will serve to inform future policy and practice in this dynamic area. It may also illuminate experiences of barriers and facilitators experienced by patients and healthcare professionals in other similar populations as there is potentially areas of convergence between pwCF and other patient populations living with physical impairments who utilise pre-conception to post-partum care.

### Patient and Public Involvement

Adults with CF who have accessed and utilised pre-conception to post-partum care and healthcare professionals with experience in delivering care to pwCF in the pre-partum to post-partum period will be involved in the interpretation and dissemination of the findings of the review. While there has been no Public and Patient Involvement in the development of this protocol, the lead author is a person with Cystic Fibrosis who has accessed and utilised pre-conception to post-partum care.

This review is one part of a larger research project. This mixed methods study will involve an empirical study exploring the experience of pre-conception to post-partum care for pwCF and the HCPs involved in their care in Ireland. 

To date the author and lead researcher has established an expert panel including pwCF from Ireland to inform the study from the protocol stages. PPI will be embedded in the study from protocol development, interview and survey guides, interpretation of findings through to dissemination stages.

## Data Availability

No underlying data are associated with this article. Open Science Framework: ‘Exploring the barriers and enablers experienced by people with Cystic Fibrosis and their healthcare professionals in accessing, utilising and delivering maternity and Cystic Fibrosis care during the pre-conception to post-partum period: A mixed methods systematic review protocol.’ https://osf.io/8fr5d/?view_only=3ca6349ea7904fff9a7cccdd844585f5 This project contains the following extended data: Sample search strategy Draft data charting template Open Science Framework: PRISMA-P checklist for ‘Exploring the barriers and enablers experienced by people with Cystic Fibrosis and their healthcare professionals in accessing, utilising and delivering maternity and Cystic Fibrosis care during the pre-conception to post-partum period: A mixed methods systematic review protocol’. https://osf.io/8fr5d/?view_only=3ca6349ea7904fff9a7cccdd844585f5 Data are available under the terms of the Creative Commons Attribution 4.0 International license (CC-BY 4.0).
