# Supplementary material for: Exploring the barriers and enablers experienced by people with Cystic Fibrosis and their healthcare professionals in accessing, utilising and delivering maternity and Cystic Fibrosis care during the pre-conception to post-partum period: A mixed methods systematic review protocol
Source: HRB Open Res. 2023 Apr 28;5:22. Originally published 2022 Mar 23. [Version 2] doi: 10.12688/hrbopenres.13500.2 (PMC10220422; doi:10.12688/hrbopenres.13500.2)
Supplement: Peer review history

# Peer review: Reviewer response for version 2

**DOI:** 10.21956/hrbopenres.14992.r33695

**Published:** 2023-05-26

**Authors:** Lyndsay A. Alexander

**Affiliations:** 1 School of Health Sciences, Robert Gordon University, Aberdeen, UK

The author response is clear and they have addressed the previous comments fully. I have no other comments to add and look forward to reading the results of this review.

Is the study design appropriate for the research question?

Yes

Is the rationale for, and objectives of, the study clearly described?

Yes

Are sufficient details of the methods provided to allow replication by others?

Partly

Are the datasets clearly presented in a useable and accessible format?

Not applicable

Reviewer Expertise:

methodology in evidence synthesis

I confirm that I have read this submission and believe that I have an appropriate level of expertise to confirm that it is of an acceptable scientific standard.

# Peer review: Reviewer response for version 2

**DOI:** 10.21956/hrbopenres.14992.r33696

**Published:** 2023-05-09

**Authors:** Emily M. Godfrey

**Affiliations:** 1 Department of Family Medicine, University of Washington, Seattle, WA, USA

I appreciate the thoughtful reply by the authors. They have done a nice job responding to the reviewer's comments.

 MMAT is an excellent choice as the data extraction tool for this mix methods systematic review. The authors may want to specifically mention whether case reports will be excluded from this review.  Generally speaking its pretty rare for authors who published a study (sometimes years ago) to respond to request for missing or extra data.  What will the authors of this systematic review do if data is not acquired from authors whom they try to contact?

 Otherwise, I have no further comments and wish the authors the best success in completing this systematic review.

Is the study design appropriate for the research question?

Yes

Is the rationale for, and objectives of, the study clearly described?

Partly

Are sufficient details of the methods provided to allow replication by others?

Partly

Are the datasets clearly presented in a useable and accessible format?

Not applicable

Reviewer Expertise:

Sexual and reproductive health in cystic fibrosis, patient engagement research methods, systematic reviews

I confirm that I have read this submission and believe that I have an appropriate level of expertise to confirm that it is of an acceptable scientific standard.

# Peer review: Reviewer response for version 1

**DOI:** 10.21956/hrbopenres.14725.r32993

**Published:** 2022-10-25

**Authors:** Emily M. Godfrey

**Affiliations:** 1 Department of Family Medicine, University of Washington, Seattle, WA, USA

The authors are presenting an important and pressing question for pwCF and the findings will likely be meaningful for investigators who wish to bolster sexual and reproductive health in CF care. They did a nice job with this protocol in terms of thinking through the PICOS question, search strategy, study selection, assessing quality, and synthesis integration. I see some issues that may arise with performing this systematic review.

 I think the authors could better justify their rationale for and define their broad outcomes under barriers and enablers. They mention looking at these outcomes at macro, mesa and micro levels, but what is meant by that is unclear. It seems that if barriers include constructs, attitudes, perceived challenge that such outcomes represent more of a micro level, as opposed to something more macro, like the health system or how care is paid for?

 I appreciate that the authors have patient and public involvement. I encourage participants that represent different health systems globally, as access to care differs from country to country. The authors should also be aware that there are initiatives coming from pwCF who have done work in this area and whose publications may not show up under the proposed search strategy. Here is an initiative by pwCF: https://cfreshc.org/srh-guide/

 Is there an opportunity to also include reports from the grey literature (such as CF Roundtable) – pwCF-driven newsletter whereby pwCF report their experiences (with stories over the past year that specifically include SRH stories) that may be enlightening in terms of fully understanding the barriers and enablers to care in this arena? 

- Sufian S, Mentch L and Godfrey EM. Women with CF are Helping Guide Reproductive and Sexual Health Research. CF Roundtable: A newsletter for adults who have Cystic Fibrosis, Summer 2017; 27(3): 16-17, 28
- Pam, M. What is a family? Why does it matter? CF Roundtable: A newsletter for adults who have Cystic Fibrosis, Summer 2020; 30(3):10-11

Is the study design appropriate for the research question?

Yes

Is the rationale for, and objectives of, the study clearly described?

Partly

Are sufficient details of the methods provided to allow replication by others?

Partly

Are the datasets clearly presented in a useable and accessible format?

Not applicable

Reviewer Expertise:

Sexual and reproductive health in cystic fibrosis, contraception abortion, patient engagement methods, systematic reviews

I confirm that I have read this submission and believe that I have an appropriate level of expertise to confirm that it is of an acceptable scientific standard, however I have significant reservations, as outlined above.

# Response

**Published:** 2023-04-24

**Authors:** Jen Balfe

**Affiliations:** Royal College of Surgeons in Ireland, Ireland

Thank you very much for taking the time to read and respond to our systematic review protocol. We appreciate your constructive and considered feedback. We have addressed your suggestions and advice in our revised manuscript.

 1) Thank you for highlighting any lack of clarity here in terms of the outcomes relating to barriers and enablers. The authors were informed by the approach of Smith et al., (2019) who, although focusing on the Health Care Professional perspective provide an adaptable and employable set of definitions for the three stages that will be addressed in this study. Their definitions and our intent to adapt them accordingly for our study is outlined now on page 5 paragraph 4.

 2) The authors are aware of the excellent and collaborative initiatives by CFRESHC and others and considered at the early stages of the protocol development including such resources.

 From an initial scoping review, the lack of peer reviewed articles on experience in comparison to the amount of peer reviewed articles on outcomes and guidelines, highlighted the need to conduct this review.

 This review is one part of a larger research project which was co-funded by the Irish Research Council and Cystic Fibrosis Ireland. Another arm of the project will involve an empirical study exploring the experience of pre-conception to post-partum care for pwCF and the HCPs involved in their care in Ireland.

 To date the lead author and lead researcher has established an expert panel including pwCF from Ireland to inform the mixed-methods study from the protocol stage. Public and Patient Involvement(PPI) will be embedded in the study from protocol development, interview and survey guides, interpretation of findings through to dissemination stages.

 This information is now included in the section referring to Public and Patient Involvement on page 9.

 In order to ensure that the scope of the review was not too broad as to make it unachievable within the time frame to feed into the empirical study, it was decided that the primary focus would be on peer reviewed empirical studies. When disseminating the results of the findings of the review, the authors will ensure they share their findings with the global CF community through social media and publications/presentations.

 3) As outlined above it was felt that it was important to assess the peer reviewed and empirical literature first so as to establish the context of fertility and pregnancy in CF in this sphere. Following the JBI approach we will use critical appraisal tools to assess the current literature pertaining to the topic.

 The authors agree that an exploration of grey literature and its potential to illuminate barriers and enablers as considered in this area is a worthwhile future study.

# Peer review: Reviewer response for version 1

**DOI:** 10.21956/hrbopenres.14725.r31872

**Published:** 2022-06-06

**Authors:** Lyndsay A. Alexander

**Affiliations:** 1 School of Health Sciences, Robert Gordon University, Aberdeen, UK

Thank you for the invitation to review this protocol.

 There has been a clear rationale and need for this review presented although I have a couple of methodological questions related to the protocol for clarity:

1. I may have missed it but which languages will be included in this review? As international evidence will be included, will there also be all languages? if so, what is the plan for translation?
2. Please clarify which version of the Covidence data extraction software will be used and also provide some more detail on how the qualitative data will be assessed by theme and categorised, and how this relates to the JBI mixed methodology.
3. The draft data extraction chart is referred to but are there any plans for piloting to establish reliability between reviewers extracting data and also how to resolve any conflicts? how many reviewers will be involved in the synthesis?
4. As JBI does not have a Mixed method critical appraisal tool, can the authors clarify what JBI CA tool they are referring to for this study design? 
5. Greater detail for the recruitment and involvement of pwCF and HCPs delivering care that will be involved in the interpretation and dissemination of the review findings are required as this could not be replicated as presented and could be considered tokenistic.
6. Although the protocol has been published on OSF, the draft search strategy and data extraction chart should be included as an appendix here for full transparency and peer review. I am unable to review them here due to this. 

Is the study design appropriate for the research question?

Yes

Is the rationale for, and objectives of, the study clearly described?

Yes

Are sufficient details of the methods provided to allow replication by others?

Partly

Are the datasets clearly presented in a useable and accessible format?

Not applicable

Reviewer Expertise:

methodology in evidence synthesis

I confirm that I have read this submission and believe that I have an appropriate level of expertise to confirm that it is of an acceptable scientific standard, however I have significant reservations, as outlined above.

# Response

**Published:** 2023-04-24

**Authors:** Jen Balfe

**Affiliations:** Royal College of Surgeons in Ireland, Ireland

Thank you very much for taking the time to read and respond to our systematic review protocol. We appreciate your constructive and considered feedback. We have addressed your suggestions and advice in our revised manuscript.

 1) Due to constraints on time and resources the review will focus only on English language studies. Given the sometimes complex and medical nature of the expected articles it was considered best not to rely on Google translate for accuracy purposes especially when quotes from participants in studies being reviewed are to be included. The JBI approach is to be as inclusive as possible and the authors recognise that not including languages will be a limitation of the study, and this will be highlighted when findings are being written up.

 2) Covidence 2.0 software will be used. In keeping with the JBI approach to extracting findings, the data extraction will follow two phases. Study characteristics such as country, setting, stage of care and population will be included. To this we have added main results, evidence gaps and recommendations and this is included now on page 8, paragraph 1 under the heading Data Extraction.

 In the second phase, findings will be extracted. Findings will be extracted alongside illustrations/quotes from the text that demonstrate where the finding originated. Findings will be coded, after which themes will be developed. As noted on page 8 under Data Extraction two independent reviewers will be involved in coding, with disputes being reached by consensus or with a third reviewer.

 The findings and illustrations will be extracted verbatim from the paper completely unedited. Illustrations may include supporting data, fieldwork observations or verbatim words of participants.

 On page 8 under ‘Data Synthesis and Integration’ the process of combining the qualitative data with the qualitized data into categories is detailed further.

 3) Yes there are plans to pilot the data extraction sheet to establish reliability. A sample of five articles which have progressed to full text screening will be piloted by two reviewers. We have added this, page 8, paragraphs 2 and 3.

 The lead author will review all articles and two other reviewers will sample half the texts each. The data extraction template will be completed by two reviewers. The piloted extraction sheets will be reviewed by the author and two reviewers and any conflicts will be resolved by consensus or by decision of a third reviewer. This is now added to page 8 paragraph 3.

 Two authors will be involved in the synthesis of findings. Any conflicts will be involved by a consensus agreement or in consultation with a third reviewer.

 4) The authors intend to use the Mixed Methods Appraisal Tool (MMAT) for the critical appraisal of this study design. This is now included in the section ‘Assessment of methodological quality’.

 5) A PPI panel of pwCF and HCPs and other stakeholders will be consulted in the interpretation of the findings. CF Ireland will use their social media accounts to raise awareness and invite discussion on the findings.

 One author on this study is a HCP with expertise in delivering care to pwCF during pregnancy, the lead author is a pwCF who has been pregnant twice. A third author is the senior research and policy coordinator with the charity and advocacy organisation Cystic Fibrosis Ireland. All three authors have informally consulted with other stakeholders in the field, including pwCF, HCPS and charity representative bodies.

 As a pwCF the author is deeply aware of the importance of participation and collaboration between HCPs and pwCF to further the research aims of pwCF and hopes that in doing so she will elucidate the potential for co-creation and design of research in a way that is fully participatory and engaged and moves beyond the trope of tokenism.

 6) As stipulated on the HRB Open Research guidelines pertaining to supplementary material, ‘all materials associated with a manuscript are visible, FAIR, and subject to peer review, HRB Open Research does not accept supplementary material. Additional materials that support the key claims in the paper but are not absolutely required to follow the study design and analysis of the results, e.g. questionnaires, or supporting images or tables, can be included as extended data. Extended data should be deposited in an approved repository and listed as part of the data availability statement.’ As such the search strategy and data extraction sheet are published on OSF.
